# Effect of black ginseng and silkworm supplementation on obesity, the transcriptome, and the gut microbiome of diet-induced overweight dogs

**DOI:** 10.1038/s41598-021-95789-8

**Published:** 2021-08-11

**Authors:** Miey Park, Ki Hyun Kim, Varun Jaiswal, Jihee Choi, Ju Lan Chun, Kang Min Seo, Mi-Jin Lee, Hae-Jeung Lee

**Affiliations:** 1 Department of Food and Nutrition, College of BioNano Technology, Gachon University, 1342 Seongnam-daero, Sujeong-gu, Seongnam-si, 13120 Gyeonggi-do Korea; 2 Animal Welfare Research Team, National Institute of Animal Science, National Institute of Animal Science, Rural Development Administration, Wanju, 55365 Korea; 3 Clinical Nutritional Medicine, Veterinary Medical Teaching Hospital, Seoul National University, Gwanak-ro, Gwanak-gu, Seoul, 08826 Korea

**Keywords:** Genetics, Microbiology, Molecular medicine

## Abstract

Like humans, weight control in overweight dogs is associated with a longer life expectancy and a healthier life. Dietary supplements are one of the best strategies for controlling obesity and obesity-associated diseases. This study was conducted to assess the potential of black ginseng (BG) and silkworm (SW) as supplements for weight control in diet-induced overweight beagle dogs. To investigate the changes that occur in dogs administered the supplements, different obesity-related parameters, such as body condition score (BCS), blood fatty acid profile, transcriptome, and microbiome, were assessed in high energy diet (HD) and HD with BG + SW supplementation (HDT) groups of test animals. After 12 weeks of BG + SW supplementation, total cholesterol and triglyceride levels were reduced in the HDT group. In the transcriptome analysis, nine genes (*NUGGC*, *EFR3B*, *RTP4*, *ACAN*, *HOXC4*, *IL17RB*, *SOX13*, *SLC18A2*, and *SOX4*) that are known to be associated with obesity were found to be differentially expressed between the ND (normal diet) and HD groups as well as the HD and HDT groups. Significant changes in some taxa were observed between the HD and ND groups. These data suggest that the BG + SW supplement could be developed as dietary interventions against diet-induced obesity, and obesity-related differential genes could be important candidates in the mechanism of the anti-obesity effects of the BG + SW supplement.

## Introduction

Increased food availability, excessive nutrient intake, and reduced movement are known to increase the risk of obesity and related diseases, such as diabetes and metabolic syndrome^1^. Many studies have demonstrated that the organs affected by metabolic syndrome contain excess levels of triglycerides^2–4^. Lipids have fatty acids (FAs) as part of their structure, which play a variety of biological roles in health maintenance and function as signaling molecules^5^. Imbalance in lipid signaling pathways can lead to allergy, autoimmunity, chronic inflammation, and metabolic and degenerative diseases^6^. Bioactive lipids are involved in various inflammatory processes and modulate immune cell function to regulate the wide range of responses that induce the pathologies in metabolic-related diseases^7^.

Body condition score (BCS) is a semi-quantitative, straightforward method of assessing body fat composition that divides the continuum of superficial body composition into a finite number of ordered categories^8^. BCS can also be used as an alternative tool for predicting specific diseases^9^. The BCS system for dogs is based on a nine-point scale; an ideal BCS is 4–5, 6–7 is overweight, and a score > 8 is obese^10^. Numerous factors can cause obesity, including genetics, the amount of physical activity, and the energy content of the diet^11^. The prevalence of overweight and obesity among dogs is 33.5% and 7.6%, respectively, and the prevalence increases with age, up to about 10 years old^12^. Transcriptome profiling of obese and insulin-resistant mice highlighted differences in signaling, lipid metabolism, and inflammation^13,14^. Metabolomics and microbiome analysis have shown the potential to evaluate changes in metabolic states^15,16^, and the identification of metabolic patterns associated with obesity might be useful in preventing metabolic-related diseases and even cancer^17^.

Multiple studies have shown the effects of diet on the gut microbiota, and the gut microbiota has been described as an essential contributor to the development of obesity-related metabolic dysfunction^18–22^. A high-fat diet (HFD) can alter the dominant gut microbiota, and its metabolites^23^. Prolonged HFD feeding in mice resulted in significant changes in the intestinal microbiome and brain metabolites, induced depression-like behavior, and reduced the abundance of the phylum *Bacteroidetes* in the intestine of depressed subjects^24^.

Ginseng has been used as a dietary supplement and has been shown to have therapeutic effects against obesity, cancer, and cardiovascular disease and improve immune and cognitive function^25,26^. Previous studies have also reported that ginseng saponin inhibits pancreatic lipase activity in vitro, and ginsenoside plays a significant role in the antidiabetic effects of ginseng in obese diabetic mice^27,28^. Orally administrated compound K suppressed the elevation of plasma triglyceride in the dog^29^. Black ginseng (BG) is prepared from raw white ginseng by steaming and drying it nine times. This process turns it black, which is accompanied by chemical changes in secondary metabolites. After processing, black ginseng contains more secondary metabolites than other ginseng^30^. Based on its pharmacological effects, BG has been shown to possess higher biological activity levels than red ginseng^30^. Although BG has been shown to be effective for obesity, the anti-obesity effect of BG in canines has not yet been investigated.

Recently, the silkworm (SW) *Bombyx mori* has received increased scientific attention, as several studies have reported its beneficial effects against liver damage^31,32^, hyperglycemia^33^, type 2 diabetes^34^, and Parkinson’s disease^35^. In addition, 1-deoxynojirimycin (1-DNJ), a potent α-glucosidase inhibitor found in mulberry leaves and silkworms, was shown to possess anti-obesity, anti-hyperglycemic, and anti-tumor effects^36^. Silkworms are used as food, and they are an essential source of protein in some mountainous regions in Asia^37^. Compared to soy proteins, silkworm proteins have higher or similar levels of essential amino acids, except isoleucine and leucine, and diverse fatty acids, such as palmitic acid, oleic acid, linoleic acid, and stearic acid^37^. Treatment of rats with silkworm oil for 18 weeks significantly reduced total cholesterol and triglyceride levels in the blood and markedly increased high-density lipoprotein cholesterol^38^. Moreover, no hepatotoxicity or mutation has been reported following ingestion of silkworms^39,40^. In addition, SW treatment significantly induced phosphorylation of AMPK and ACC in obesity mouse liver. AMPK activation is an essential process to inhibits the FAS protein level and activation of SREBP-1c. SW showed anti-obesity effects in a mouse model^41^.

Our previous studies reported that the BG and ginsenoside Rb1 promote the browning effect by inducing UCP1 expression in white adipocytes^42^. Also the BG and SW ameliorated nonalcoholic fatty liver disease in free fatty acid-induced liver cells and high-fat/high-fructose diet mice^41,43^. We would like to confirm the anti-obesity effects of BG and SW treatment in dog models. This study aimed to evaluate the impacts of BG and SW supplementation in weight control for 16 weeks in overweight beagle dogs by analyzing the serum fatty acid profiles, RNA expression in whole blood, and gut microbiome, which is involved in energy metabolism.

## Materials and methods

### Animals and diet

Nine (five males and four females) healthy, spayed and neutered 22-month-old beagle dogs (body weight [BW] 8.4 ± 0.61 kg and BCS 4.2 ± 0.17) were used in this study. All dogs were housed under controlled environmental conditions and were professionally supervised at the National Institute of Animal Science (Wanju-gun, Jeollabuk-do, Korea) of the Rural Development Administration during the study. Ethical approval for this study (NIAS-2019-370) was obtained, and the study was conducted in accordance with the guidelines of the Animal Care and Use Committee of the National Institute of Animal Science. After 6 months of acclimatization, the dogs were randomly divided into two groups: the normal diet group (ND; optimal energy intake) and the high energy intake group (HD). After a 4-week high-energy induction period, the beagle dogs were randomly divided into three groups: the normal diet (ND: optimal energy intake) + placebo, HD (high energy intake) + placebo, and HDT (high energy intake + 100 mg·kg^−1^·day^−1^ black ginseng [BG (Table 1)]^43^ + 100 mg·kg^−1^·day^-1^ silkworm powder [SM (Table 2)])^41^ for 12 weeks. All dogs were housed under similar conditions and fed basic feed once a day (at 10:00 am) that is fully balanced and meets the nutritional requirements of dogs (Iskhan All-life33^®^; Wooriwa Ltd., Korea; 4,100 kcal/kg metabolizable energy, > 33% protein, > 20% fat, > 19% carbohydrates, and < 12% moisture) as suggested by the Association of American Feed Control Officials (AAFCO, 2018). The HD group was provided additional wet feed (ZIWI® peak Tripe & Lamb recipe, Ziwi Ltd., New Zealand; 1,150 kcal/kg metabolizable energy, > 9% protein, > 4% fat, > 5% carbohydrates, and < 78% moisture), which was equivalent to 20% of the energy consumed from basic food. The metabolic energy requirements (MER) of dogs were calculated by AAFCO's MER calculation method following as; MER = 132 × metabolic body weight (mBW). The BG and SW supplements were soft encapsulated and fed to the dogs once a day with the morning feed (at 10:00 am). Maltodextrin (1000 mg) was used as a placebo and was also soft encapsulated and fed to the ND and HD groups once a day with the morning feed. All dogs had free access to water and ran outside for several hours each day throughout the study period. Each experimental dog was placed in a separate breeding space (1.8 m × 2.6 m) of the facility and raised under controlled conditions at 22–24 °C, 60–80% humidity, and a 12-h light–dark cycle. Food intake was measured daily, and the BCS was evaluated once a week for 16 weeks using a 9-point scale based on the criteria of Laplamme et al.^8^.

**Table 1:** Composition of ginsenosides in black ginseng (BG).

Ginsenosides	BG, Root (mg/g)
Rg1	0.12
Re	0.21
Rf	0.64
Rh1(S)	0.84
Rg2(S)	1.16
Rg2(R)	0.64
Rh1(R)	0.41
Rb1	3.11
Rc	1.56
F1	0.00
Rb2	1.99
Rb3	0.30
Rd	0.98
F2	3.56
Rg3(S)	1.78
Rg3(R)	1.36
PPT(S)	0.00
PPT(R)	0.00
K	2.34
Rh2(S)	0.00
Rh2(R)	0.00
Total*	21.00

*Sum of individual ginsenoside content.

**Table 2:** Metabolites identified in a silkworm powder (SW).

No	Tentative identification *	Unique mass (*m/z*)	ID !	No	Tentative identification	Unique mass (*m/z*)	ID
**Amino acids**				**Etc**			
1	Valine	144	STD MS @	22	Butanediol	117	MS
2	Serine	204	STD	23	Hydroxylamine	133	MS
3	Threonine	117	STD	24	Pyruvic acid	133	MS
4	β-Alanine	174	MS	25	Urea	189	MS
5	Aspartic acid	232	STD MS	26	Hydroxybenzoic acid	267	STD MS
6	Pyroglutamic acid	156	MS	27	α-Glycerophosphoric acid	299	MS
7	Glutamic acid	246	MS	28	1-Deoxynojirimycin	420	STD
8	Asparagine	116	STD MS	29	Pantothenic acid	291	MS
9	Lysine	156	STD MS	30	Phytol	143	MS
10	Tyrosine	218	STD MS	**Non-identifications**			
**Sugar and sugar derivatives**				31	N.I. 1	171	‒
11	Glyceric acid	189	MS	32	N.I. 2	89	‒
12	Carbohydrate 1	103	MS	33	N.I. 3	123	‒
13	Carbohydrate 2	205	MS	34	N.I. 4	86	‒
14	Ribonic acid	103	MS	35	N.I. 5	86	‒
15	D-Glucose	205	STD MS	36	N.I. 6	57	‒
16	Carbohydrate 3	319	MS	37	N.I. 7	84	‒
17	myo-Inositol	217	STD MS	38	N.I. 8	131	‒
18	Carbohydrate 4	319	MS	39	N.I. 9	205	‒
19	Glyceryl-glycoside	204	MS	40	N.I. 10	117	‒
**Fatty acids**				41	N.I. 11	129	‒
20	Stearic acid	117	STD MS	42	N.I. 12	245	‒
21	Oleamide	131	MS	43	N.I. 15	129	‒

*Tentative metabolites based on variable important projection (VIP) analysis with a cutoff value of 0.7 and p-value < 0.05; ! Identification: STD, Standard

@MS fragment patterns detected.

### Blood sampling, serum fatty acid profile analysis, and fecal sampling

Blood samples were collected from the dogs at treatment initiation and after 4, 8, and 12 weeks. Blood was collected into a tube, left for more than 30 min, and then centrifuged at 400 × *g* for 10 min at 4 °C. The serum was stored at − 80 °C until use. Blood samples were also collected at the end of the experiment (after 12 weeks of supplementation) for RNA-Seq analysis. Whole blood (500 μl) was collected from the dogs into RNAprotect® Animal Blood Tubes (QIAGEN, Hilden, Germany) and was stored at − 80 °C until use. Total RNA was purified from thawed RNA-preserved blood using the RNeasy Protect Animal Blood Kit (QIAGEN, Hilden, Germany) according to the manufacturer’s guidelines. Total cholesterol (TC) and triglyceride (TG) levels were analyzed using commercial kits according to the manufacturer’s protocols. Fecal samples were collected at the end of treatment and stored at − 80 °C until use.

### RNA processing and sequencing

Total RNA (500 ng) was used to prepare whole transcriptome sequencing libraries. Whole transcriptome RNA was enriched by depleting ribosomal RNA (rRNA), and a complete transcriptome sequencing library was generated using the MGIEasy RNA Directional Library Prep Kit (MGI) according to the manufacturer’s instructions. After rRNA depletion, the remaining RNA was fragmented into small pieces by treatment with divalent cations under elevated temperatures. Then, the cleaved RNA fragments were copied into first-strand cDNA using reverse transcriptase and random primers. Strand specificity was achieved in RT directional buffer, followed by second-strand cDNA synthesis. An additional A base was added to the cDNA fragments and then an adapter was ligated. The products were then purified and enriched by PCR to create the final cDNA library.

The double-stranded library was quantified using the QauntiFluor ONE dsDNA System (Promega, Madison, WI, USA) and 330 ng in a total volume of 60 μl or less. The library was cyclized at 37 °C for 60 min, digested at 37 °C for 30 min, and then the circularization product was cleaned up. The library was incubated at 30 °C for 25 min with DNA nanoball (DNB) enzyme. Finally, the library was quantified using the QauntiFluor ssDNA System (Promega) and sequenced using the MGIseq system (MGI) to generate 150 bp paired-end reads.

### RNA-Seq pipeline for assembly and differential expression analysis

The NGS system generated pair-end reads as output, which were subjected to quality control using AfterQC^44^. Filtering, trimming, and error removal were performed with default parameters to obtain high quality reads. These reads were aligned with the reference genome using HISAT2^45^. The dog (*Canis lupus familiaris*) CanFam3.1 reference genome assembly released by The Genome Reference Consortium was used for the alignment of reads from all samples, and the resulting alignment files were saved as Sequence Alignment Map (SAM) files. The SAM files were sorted and converted to BAM files using SAMtool^46^. The BAM files were used for the assembly analysis using StringTie with the -e option to combine the assembly results of all nine samples for differential expression analysis^47^. A Python script (prepDE.py) was used to combine the assembly results for all nine samples. Finally, for the expression analysis, a matrix consisting of the read count values, corresponding to every assembled gene/transcript, was created. The gene expression of all samples, in the form of a gene count table, was used in iDEP.92^48^ for differential expression-related analyses using DESeq2 and EdgeR. In the analysis, the default threshold false discovery rate (FDR), < 0.1, and a minimum fold change of 2 were used to identify the differentially expressed genes (DEGs).

### Functional enrichment of differentially expressed genes

The DEGs identified from the three main comparisons (ND versus HD, ND versus HDT, and HD versus HDT) were subjected to functional enrichment analysis through PANTHER^49^ (Protein ANalysis THrough Evolutionary Relationships) using *Canis lupus familiaris* as the reference organism for the analysis. The PANTHER provides results based on biological process, cellular component, molecular function, protein class, and pathway of the given genes, which can be stored as figures and excel tables.

### Identification of the important DEGs

Common DEGs in different comparisons were identified through subset analysis using InteractiVenn^50^. Finally, the DEGs that had contrasting differential expression when comparing ND and HDT with HD, that is, genes that were upregulated in the HD group when compared to the ND group and downregulated in the HDT group when compared to the HDT group, were identified. Similarly, genes that were downregulated the HD group when compared to the ND group and upregulated in the HDT group when compared to the HD group.

### Protein–protein interaction networks

The protein–protein interaction network was analyzed using STRING version-11^51^. The Ensembl IDs of the DEGs were used to generate a protein–protein interaction network using *Canis lupus* as the reference organism.

### Quantitative real time PCR analysis

Total RNA was isolated from whole blood using an RNA extraction kit (iNtRON Biotechnology, Gyeonggi-do, Korea). RNA (50 ng) was reverse transcribed to cDNA using the iScript cDNA synthesis kit (BioRad, Hercules, CA, USA). Real-time PCR was performed with TB Green Master Mix (TaKaRa Bio, Otsu, Japan) and was analyzed using QuantStudio 3 (Thermo Fisher Scientific, San Jose, CA, USA). The primer sequences used for PCR are shown in Supplementary Table 1 and were normalized to β-actin.

### Microbiome analysis

Fecal DNA was extracted from 180–220 mg samples of feces using the NucleoSpin® DNA Stool Kit (Macherey–Nagel, Germany) according to the manufacturer’s guidelines. The sequences of the 16 s rDNA V3 and V4 hypervariable regions were amplified according to the manufacturer’s instructions. The following primers were used to amplify the V3 and V4 regions: forward 5′-TCGTCGGCAGCGTCAGATGTGTATAAGAGACAGCCTACGGGNGGCWGCAG-3′ and reverse 5′-GTCTCGTGGGCTCGGAGATGTGTATAAGAGACAGGACTACHVGGGTATCTAATCC-3′. Illumina adapter overhang sequences were added to the gene-specific sequences. The locus-specific sequences were as follows: forward overhang 5′-TCGTCGGCAGCGTCAGATGTGTATAAGAGACAG-3′ and reverse overhang 5′-GTCTCGTGGGCTCGGAGATGTGTATAAGAGACAG-3′. The PCR products were purified using KAPA HiFi HotStart ReadyMix (KAPA Biosystems, USA) and an Agencourt AMPure Xp system (Beckman Coulter Genomics, USA). The libraries were sequenced on an Illumina MiSeq instrument (2 × 300 paired-end sequencing).

### Sequencing data analysis

The sequenced amplicons from selected samples were processed and analyzed using a recently updated pipeline for microbiome analysis, Quantitative Insights Into Microbial Ecology (QIIME2 version 2020.11)^52^. Pair-end reads were imported into the QIIME2 pipeline and visualized graphically for quality scoring. Divisive amplicon denoising algorithm 2 (DADA2) was used for trimming, de-noising, filter chimeras, and removing reads with low-quality scores^53^. A feature table of the amplicon sequence variants (ASVs) was constructed using downstream in QIIME2. Different cutoff values were used to trim the forward and reverse reads in order to achieve the optimal read retention count and length for DADA2 analysis. The sequences of all ASVs obtained from the DADA2 analysis were subjected to multiple sequence alignment to construct a masked, rooted phylogenetic tree using the align-to-tree-mafft-fast tree pipeline in the q2-phylogeny plugin, which uses mafft^54^ for multiple sequence alignment and FastTree^55^ to construct phylogenetic trees.

### Taxonomic annotation

Taxonomic annotation of all ASVs/features/operational taxonomic units (OTUs) was carried out using the q2-feature-classifier module of QIIME2, which uses a classify-sklearn naïve Bayes taxonomy classifier trained on the Greengenes 13_8 99% OTUs^56^. Finally, a bar plot was drawn to visualize the taxonomic annotation of each sample using the “qiime taxa barplot” module. Then, a Krona plot was created, which provides a hierarchical, interactive visualization of taxonomy^57^.

### Diversity analysis

Alpha and beta diversity metrics were calculated using the q2-diversity module. Alpha diversity metrics incorporated the information from Shannon’s diversity index (a qualitative measure of community richness), observed features (a qualitative measure of community richness), Faith’s phylogenetic diversity (a qualitative measure of community richness that incorporates phylogenetic relationships between features), and evenness (or Pielou’s evenness; a measure of community evenness). Similarly, the beta diversity metrics incorporated Jaccard distance (a qualitative measure of community dissimilarity), Bray–Curtis distance (a quantitative measure of community dissimilarity), unweighted UniFrac distance (a qualitative measure of community dissimilarity that incorporates phylogenetic relationships between features), and weighted UniFrac distance (a quantitative measure of community dissimilarity that incorporates phylogenetic relationships between features).

### Differential abundance of taxa

Lastly, to estimate and visualize the differential abundance of taxa among groups (according to treatment), linear discriminant analysis effect size (LEfSe) was used^58^. The taxonomy and ASV table results from qiime2, along with metadata information, were used to prepare the LEfSe input format file using Dokdo (https://github.com/sbslee/dokdo), and the converted input file was subjected to LEfSe analysis. Graphs depicting differences in the microbiome communities and cladograms were drawn for visualization.

### Statistical analysis

Statistical analysis was performed using GraphPad Prism 5.03 (GraphPad Software, San Diego, CA, USA) and SPSS (version 17.0). One-way ANOVA and Tukey’s post-hoc tests were used to analyze the real-time PCR results. Analysis of covariance (ANCOVA) was used to exclude gender effects, and gender factors are treated as covariates. The results of weight and BCS were analyzed as repeated-ANCOVA to assess the impact over time. And the results of the animal experiments were analyzed using two-way ANOVA and Bonferroni’s post-tests to compare replicate means by row. All data are expressed as mean ± SEM. Statistical significance was set at *P* < 0.05.

### Ethical approval

This research study was approved by the Animal Care and Use Committee of the National Institute of Animal Sciences (Ref: NIAS-2019–370), Republic of Korea. The details of the experimental designs, sampling method, feeding protocol, and criteria for the end of the humanitarian experiment (weight loss of more than 20%, change of feed and water intake, or death, etc.) were performed in accordance with relevant guidelines and regulations of the Institute. We confirming this study is reported in accordance with ARRIVE guidelines (https://arriveguidelines.org).

## Results

### Effects of black ginseng (BG) and silkworm (SW) supplements on overweight dogs

**Figure 1:**
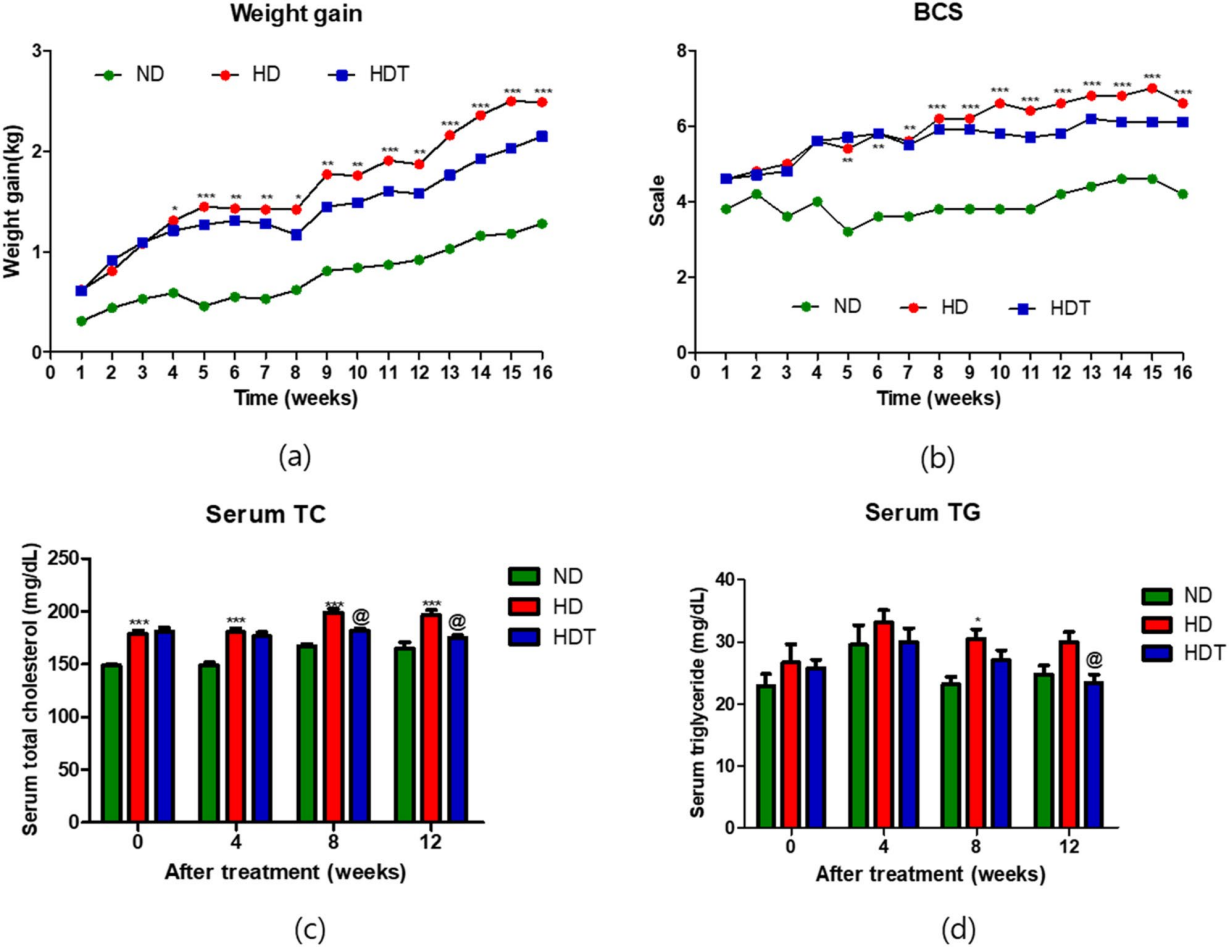
Comparison of weight gain, body condition score (BCS), serum TC, and TG in overweight beagle dogs with BG&SW supplements treatment. (**a**) Weight gain. (**b**) BCS. (**c**) Serum TC. (**d**) Serum TG. *p < 0.05, **p < 0.01, and ***p < 0.001 vs. ND. ^@^p < 0.05 vs. HD. *ND* normal diet group, *HD* high energy intake group, *HDT* high energy intake with BG&SW supplements treatment group.

The mean initial weight of the dogs in all groups was 8.4 ± 0.61 kg. After 4 weeks, significantly higher weight gains were observed in the HD group than in the ND group as a result of the additional wet feed provided to the HD group (Fig. 1a and Table 3). The BCS in the HD and HDT groups were higher than that in the ND group, and the BCS of the HD group was significantly higher than that of the ND group after 5 weeks (Fig. 1b). The increases in weight and BCS of the HDT group (BG [100 mg/kg/day] and SW [100 mg/kg/day]), were lower than those of the HD group at 4 and 8 weeks, respectively, but the differences were not significant. We evaluated serum TC and TG levels in all groups during the 12 weeks of BG + SW supplementation. Serum TC levels were significantly higher (p < 0.001) in the HD group than in the ND group. However, after BG + SW supplementation for 8 and 12 weeks, serum TC levels were significantly lower in the HDT group (p < 0.05) than in the HD group (Fig. 1c), and serum TG was significantly lower in the HDT group (p < 0.05) than in the HD group at 12 weeks (Fig. 1d).

**Table 3:** Feed and energy intake and body weight.

	ND	HD	HDT	SEM	*P*-value	
					ANCOVA	R-ANCOVA
Feed intake, g/day	221^b^	348^a^	338^a^	56	< 0.001	–
Energy intake, kcal/day	828	920	887	15	0.101	–
FCR, kg/kg	6.24	7.41	6.17	2.42	0.832	–
**Body weight**						
Initial, kg	8.31	8.24	8.51	0.61	0.672	–
Finish, kg	9.59	10.73	10.60	1.24	0.308	0.171
Change rates, %	117^b^	133^a^	125^ab^	11.5	0.169	0.045

*ND* optimal energy intake + placebo, *HD* high energy intake + Placebo, *HDT* high energy intake + 100 mg·kg^−1^·day^−1^ BG + 100 mg·kg^−1^·day^−1^ SW; *SEM* standard error mean, *ANCOVA* analysis of covariance, *R- ANCOVA* repeated-ANCOVA, *FCR* feed conversion rate, *BCS* body condition score.

^a,b^Data without same superscript in a row significantly differ (P < 0.05).

### Preprocessing and alignment of reads

To assess the effect of BG + SW supplementation on gene expression in the overweight HD and HDT groups, we carried out RNA sequencing to analyze the transcriptome profile in whole blood samples. Preprocessing and quality control were performed to obtain high quality reads for alignment with the reference genome. The high quality reads (> 98% in all samples) obtained after preprocessing, including trimming, error removal, and quality control (Supplementary Table 2), were subjected to alignment, and every read used in the alignment had a high alignment rate (average overall alignment rate > 95%) with the reference genome (Supplementary Table 2). The alignment files were used to assemble the transcripts/genes for further expression analysis.

### Transcriptome assembly and expression analysis

**Figure 2:**
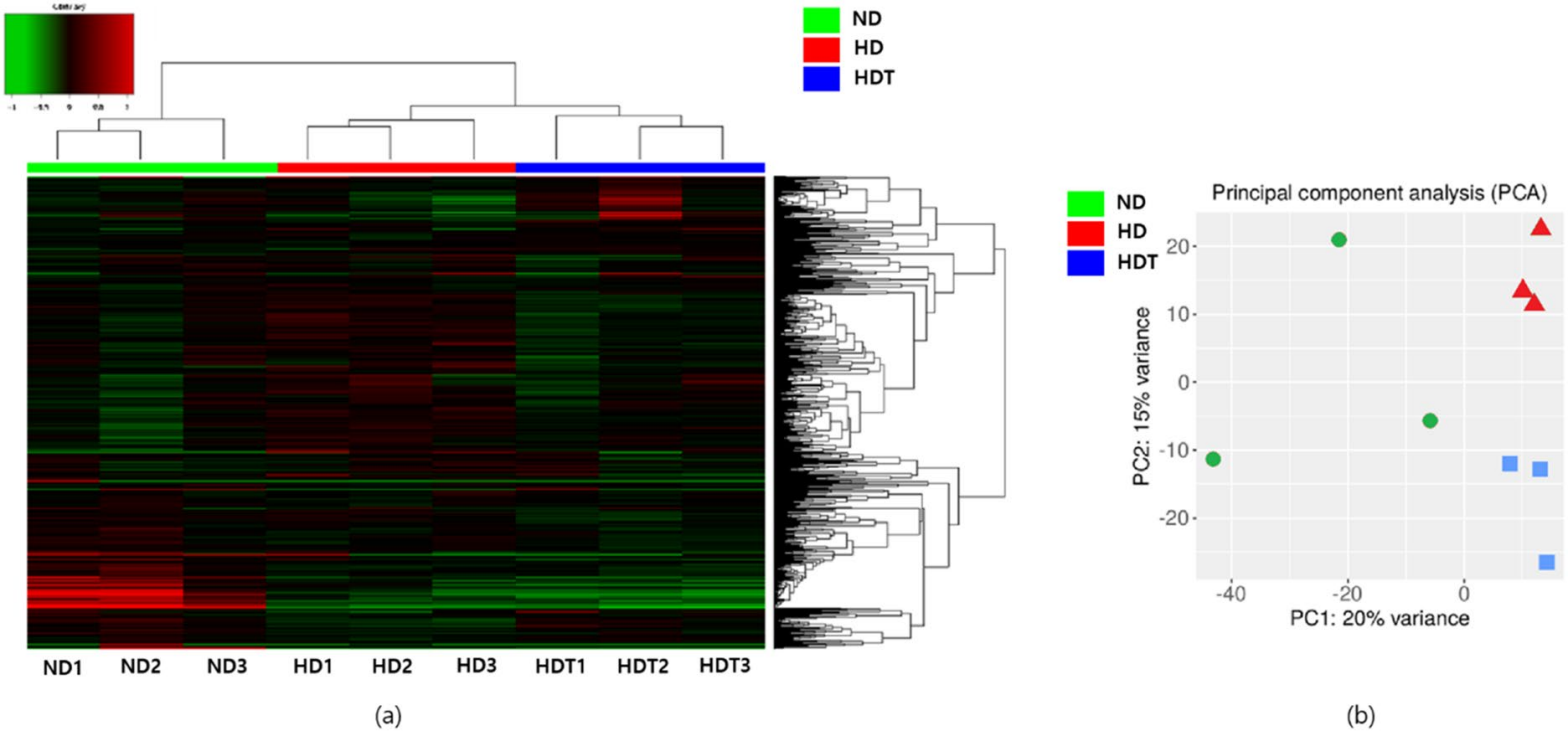
Hieradical clustering and PCA plot of differentially expressed genes. (**a**) Heat map showing hierarchical clustering of the samples on the basis of the differential expressed genes. (**b**) Principal Component Analysis (PCA) plot using the first and second principal components. *ND* normal diet group, *HD* high energy intake group, *HDT* high energy intake with BG&SW supplements treatment group.

**Figure 3:**
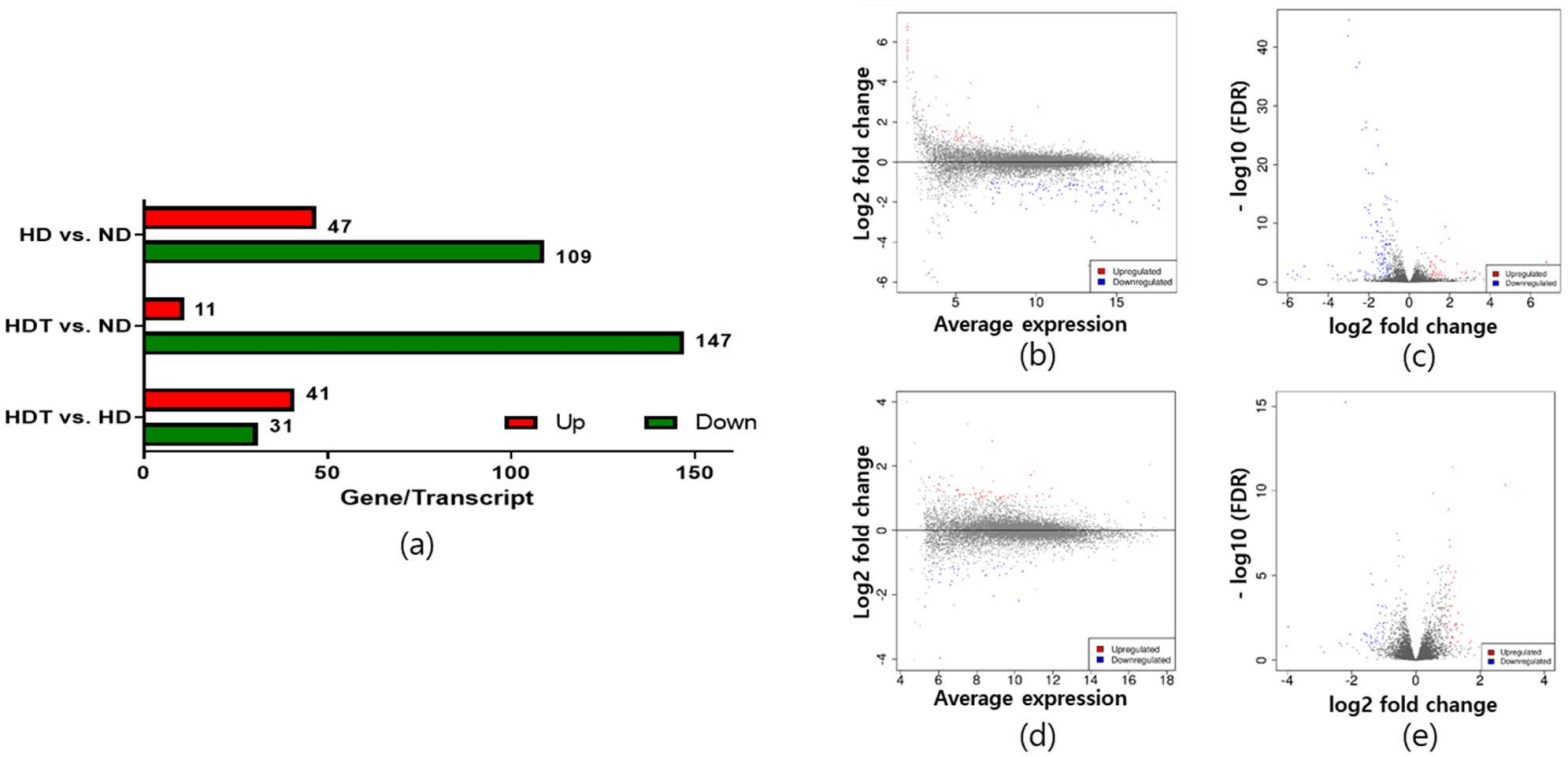
Plots to visualize the DEGs between the groups. (**a**) The number of Differentially expressed genes between all groups. (**b**) The MA plots of DEGs between the ND and the HD groups. (**c**) The Volcano plots of DEGs between ND and HD groups. (**d**) The MA plots of DEGs between the HD and the HDT groups. (**e**) The Volcano plots of DEGs between HD and HDT groups. *ND* normal diet group, *HD* high energy intake group, *HDT* high energy intake with BG&SW supplements treatment group.

The alignment was used to assemble the genes and transcripts. Then, the read counts and fragments per kilobase of transcript per million mapped reads (FPKM) for all assembled genes/transcripts were calculated. Heat maps and clustering were generated based on the differentially expressed genes. A heatmap with hierarchical clustering revealed that samples from the same group were clustered together (Fig. 2a). In the principal component analysis plot, the HD and HDT groups were clustered tightly in separate locations, but the ND group was loosely clustered in the middle of the graph (Fig. 2b). Based on a cutoff FDR of 0.1 and a minimum fold change of 2, 47, and 109 genes/transcripts were upregulated and downregulated, respectively, when the HD group was compared to the ND group. Similarly, 11 and 147 genes/transcripts were upregulated and downregulated, respectively, when the HDT group was compared with the ND group. Finally, 41 and 31 genes/transcripts were upregulated and downregulated, respectively, when the HDT group was compared to the HD group (Fig. 3a). DEGs were also plotted in MA and volcano plots to visualize the differences (Fig. 3b–e).

### Functional enrichment analysis

The upregulated and downregulated genes were subjected to functional enrichment analysis according to biological process, cellular component, molecular function, protein class, and pathway using PANTHER (protein analysis through evolutionary relationship). Most DEGs had molecular functions related to binding and catalytic activity, which can be altered by changes in obesity/fat-related mechanisms (Supplement Table 3). We compared the upregulated DEG-containing pathways between the ND and HD groups and the downregulated DEG-containing pathways between the HD and HDT groups. Ten pathways were common; these pathways were upregulated in the HD group and reduced by the BG + SW supplement in the HDT group (Supplement Table 3).

### Common DEGs in the comparison of different groups and the protein–protein interaction network

**Figure 4:**
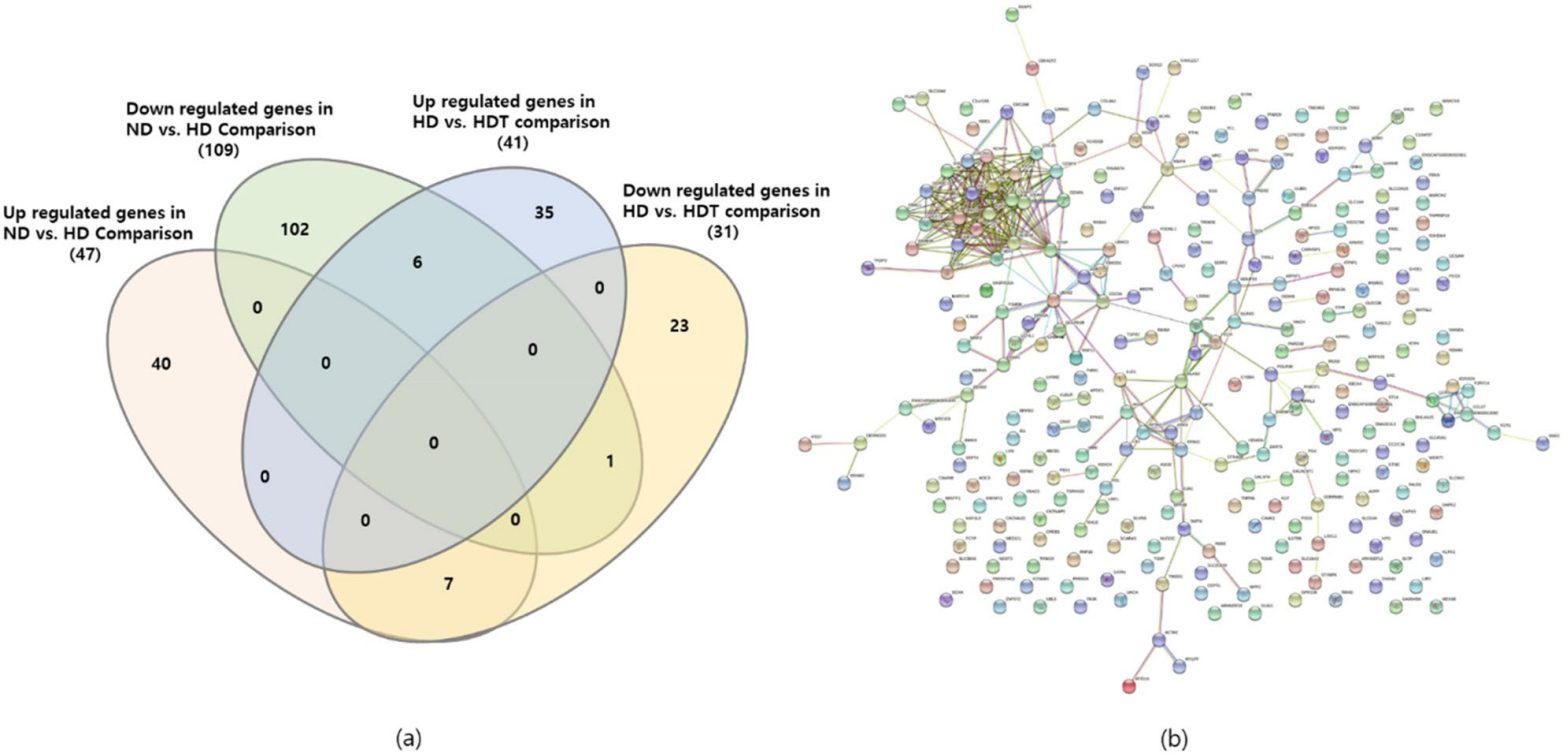
Venn diagram and protein–protein network of DEGs. (**a**) Common DEG in between ND to HD and HD to HDT groups. (**b**) Protein–protein interaction network of all DEG found in the study. *ND* normal diet group, *HD* high energy intake group, *HDT* high energy intake with BG&SW supplements treatment group.

The comparisons between the ND and HD groups and the HD and HDT groups were the most important in this study. Thus, the upregulated and downregulated DEGs were plotted in a Venn diagram, and six genes (*NUGGC*, *EFR3B*, *RTP4*, *FAM83D*, *CDC45*, and *ACAN*) were downregulated in the HD group when compared to the ND group and upregulated in the HDT group when compared to the HD group. Similarly, seven other genes (*HOXC4*, *IL17RB*, *SOX13*, *SLC30A8*, *SLC18A2*, *RHEX*, and *SOX4*) were upregulated in the HD group when compared to the ND group and downregulated in the HDT when compared to the HD groups (Fig. 4a). Finally, a protein–protein interaction network was constructed using all the DEGs using STRING version 11 (Fig. 4b). The constructed network had significantly more interactions than expected from the results, which indicated that there must be a pattern in the differentially expressed genes observed in the study.

### Validation by real-time polymerase chain reaction

**Figure 5:**
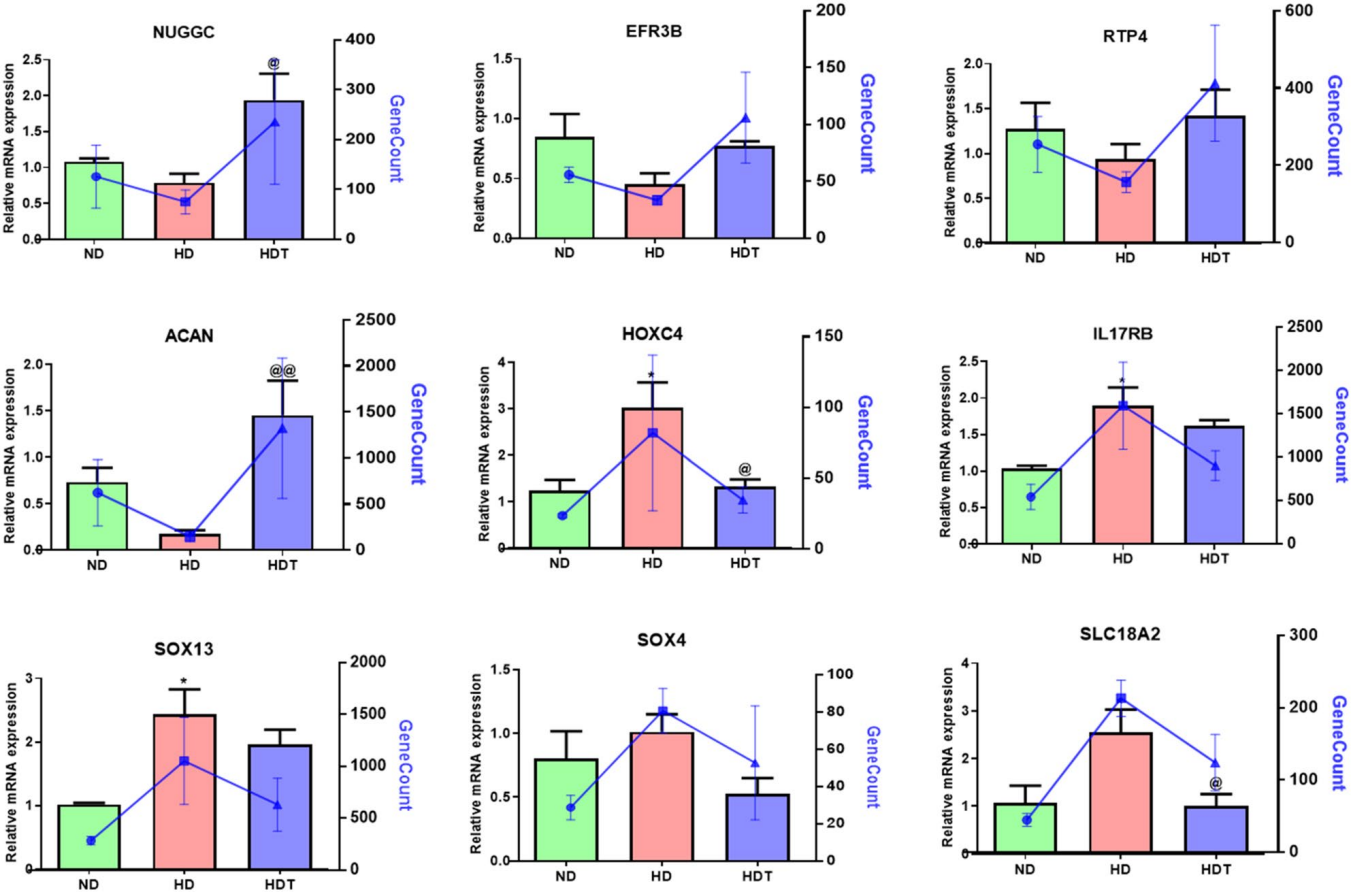
Quantitative expressions of obesity-related genes between the ND, HD, and HDT groups. * p < 0.05 vs. ND. ^@^ p < 0.05, ^@@^ p < 0.01 vs. HD. *ND* normal diet group, *HD* high energy intake group, *HDT* high energy intake with BG&SW supplements treatment group. The blue lines expressed GeneCounts between groups.

To validate the important upregulated and downregulated genes in the ND, HD, and HDT groups, we conducted a real-time polymerase chain reaction (PCR) experiment using whole blood samples from the beagle dogs. We used β-actin as a control for gene expression. The relative gene expression levels of *NUGGC* and *ACAN* were significantly higher in the HDT group than in the HD group. The expression levels of *HOXC4*, *IL17RB*, *SOX13*, and *SLC18A2* were significantly higher in the HD group than in the ND group. *HOXC4* and *SLC18A2* gene expression levels in the HDT group returned to levels similar to those in the ND group (Fig. 5).

### Gut microbiome diversity analysis

A total of nine samples, three samples from each group, were analyzed by 16S rRNA sequencing. A total of 8,880,030 pair-end reads were obtained, and after preprocessing and chimera removal using DADA2, 428,412 feature reads/operational taxonomic units (OTUs) were obtained. Finally, a total of 5,072 unique features were identified from all samples.

**Figure 6:**
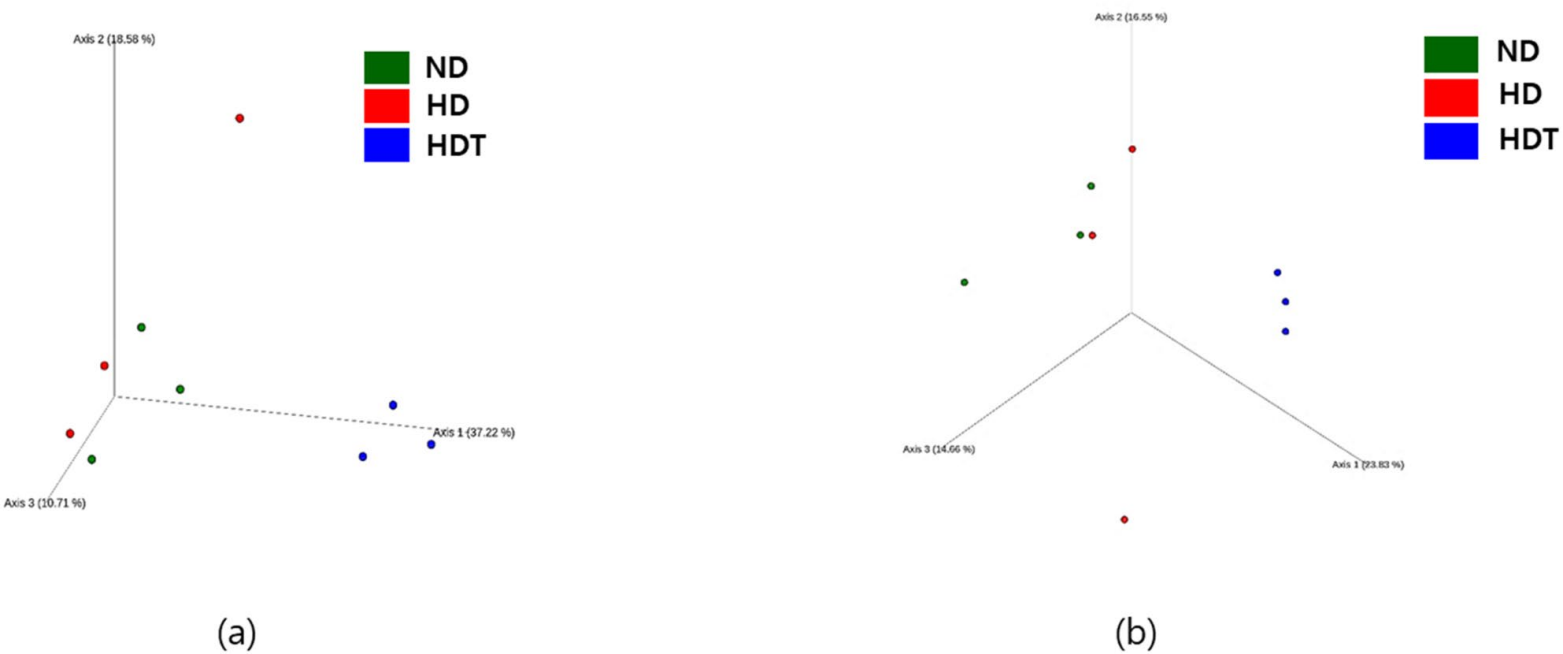
Beta diversity plots. (**a**) 3-D plot depicting beta diversity through Bray Curtis distance which is a quantitative measure of the community dissimilarity. (**b**) 3-D plot depicting beta diversity through Jaccard distance which is a qualitative measure of the community dissimilarity. *ND* normal diet group, *HD* high energy intake group, *HDT* high energy intake with BG&SW supplements treatment group.

The diversity and richness of the microbial community of the studied samples were assessed. The alpha diversity of the gut microbiome was calculated using Faith’s phylogenetic diversity and Pielou’s evenness. A significant difference (p = 0.049) in Pielou’s evenness was observed between the ND and HDT groups. No significant differences in the Faith phylogenetic diversity and Pielou’s evenness indices were observed for the other comparisons between groups. However, a decrease in Faith’s phylogenetic diversity was observed when comparing the HD group to the ND group, and a further decline was observed in the HDT group when compared to the HD group (Supplement Fig. 1b). Beta diversity analysis using Bray–Curtis distance-based principal coordinate analysis revealed that each treatment group was clustered in a separate location, although the ND and HD groups had a region of overlap, with one sample from the HD group in a distant area (Fig. 6a). A similar clustering pattern was observed in the 3D graph generated from the Jaccard distance (Fig. 6b). No significant differences were observed in the permutational multivariate analysis of variance (PERMANOVA) between the groups.

### Taxa differences among the groups

**Figure 7:**
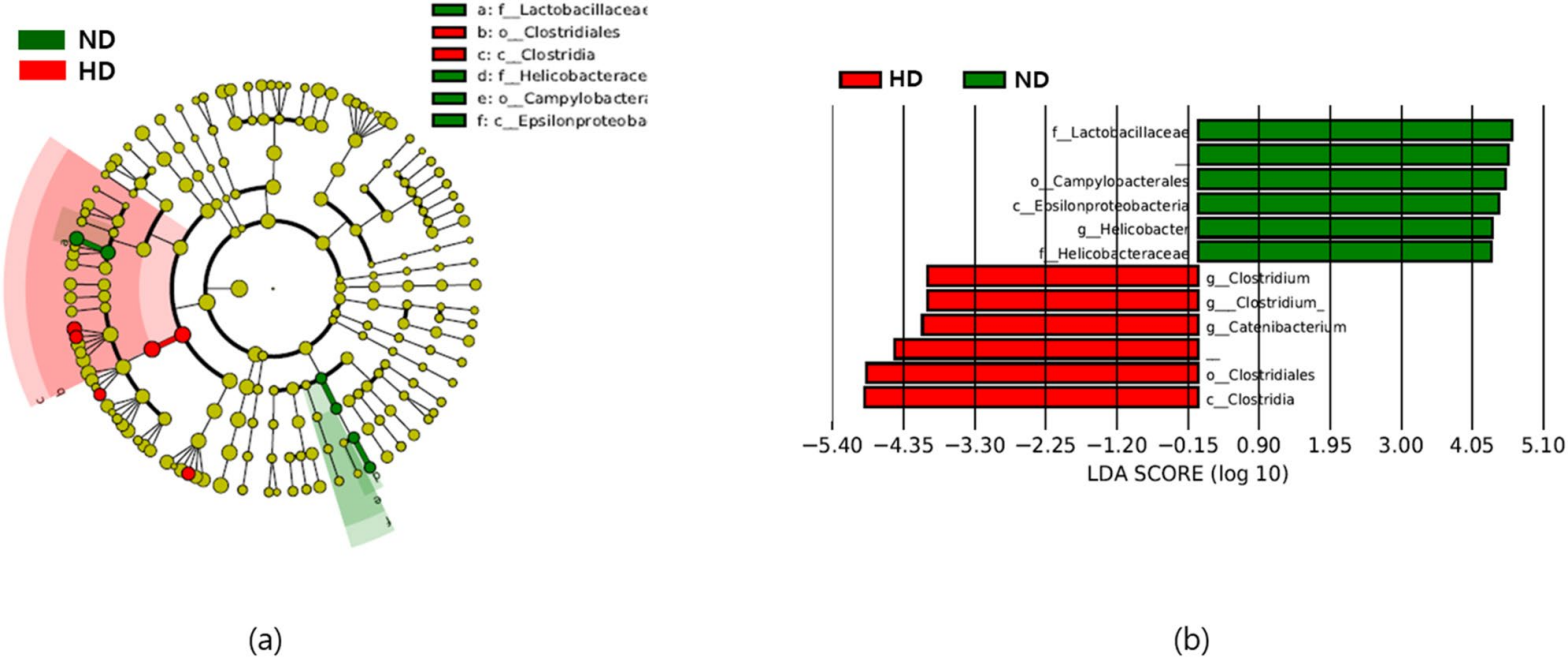
Differential abundance of taxa in ND and HD groups. (**a**) Cladogram. (**b**) Bar plot depicting bacterial taxa according to LDA. *ND* normal diet group, *HD* high energy intake group.

The taxonomy results were analyzed by generating bar plots and Krona plots to compare the significant taxa present in the gut microbiome of the different experimental groups (Supplementary Fig. 2). The most dominant phylum in all samples was Firmicutes. Other phyla, including Fusobacteria, Bacteroidetes, and Actinobacteria, were prevalent in different samples. The LEfSe analysis identified the differences in community taxa between the ND and HD groups. In the analysis, the HD group (high energy intake diet) showed an increase in the members of class Clostridia, order *Clostridiales*, genus *Catenibacterium,* and genus *Clostridium*, and decreases in the family *Helicobacteraceae*, family *Lactobacillaceae*, order *Campylobacterales*, class *Epsilonproteobacteria*, and genus *Helicobacter* (Fig. 7a,b). However, no changes in taxa were found between the HD and HDT groups based on the suggested cutoff parameter^59^.

## Discussion

Currently, the prevalence of overweight, obesity, and obesity-related metabolic dysfunction (ORMD) among dogs is elevated and is related to the presence of overweight and obesity in their owners^60,61^. There was also a positive association between BCS and the frequency of snack intake, and the BCS of a dog was greater if the owner reported that the dog consumed more snacks.

In this study, transcriptomic changes related to feeding, i.e., optimal energy intake (ND) or high energy intake (HD), and BG + SW supplementation (HDT) were examined through comparative genomic analysis of the three groups (ND, HD, and HDT). The sample quality was good, as a high alignment rate (~ 95%) with the reference genome was obtained for every sample (Supplementary Table 2). Standard methods and software were used in the RNA-Seq analysis, including AfterQC, HISAT2, StringTie, iDEP 9.2, EdgeR, and DESeq2 to identify the DEGs among the groups^45,62–64^. Pathway annotation of the DEGs yielded an interesting pattern; a total of 11 pathways that were found to be upregulated in the group administered the high energy diet were reversed/downregulated in the treated group (the group administered with black ginseng and silkworm). That is, 11 pathways (out of 16) downregulated in the HDT group when compared to HD group were upregulated in the HD group when compared to the ND group (Supplement Table 3). Based on the literature, most of these pathways (9/11), including the gonadotropin-releasing hormone receptor pathway (P06664), inflammation mediated by chemokine and cytokine signaling pathway (P00031), 5HT4 type receptor-mediated signaling pathway (P04376), Wnt signaling pathway (P00057), adrenaline and noradrenaline biosynthesis (P00001), 5HT3 type receptor-mediated signaling pathway (P04375), 5HT2 receptor-mediated signaling pathway (P04374), 5HT1 type receptor-mediated signaling pathway (P04373), and dopamine receptor-mediated signaling pathway (P05912), may be related to obesity^65–69^. These findings strongly suggest that these pathways/genes should be further examined to decipher the mechanism responsible for the anti-obesity properties of black ginseng and silkworm. Comparisons of ND with HD and HD with HDT were the most important to identify the DEGs related to the high-fat diet-induced obesity and the treatment. Four (*NUGGC*, *EFR3B*, *RTP4*, and *ACAN*) out of the six genes that were found to be downregulated in the HD group when compared to the ND group and were also upregulated in the HDT group when compared to the HD group were reported to be associated with obesity in earlier studies^70–73^. Similarly, five genes (*HOXC4*, *IL17RB*, *SOX13*, *SLC18A2*, and *SOX4*) out of seven genes that were found to be upregulated in in the HD group when compared to the ND group and downregulated in the HDT group when compared to the HD group were previously shown to be associated with obesity^74–76^. Among these obesity-associated genes, three genes (*NUGGC*, *EFR3B*, and *SOX4*) were found to be upregulated or downregulated according to the presence of obesity both in the literature and in the current study^70,71,77^. Interestingly, one gene that corresponds to microRNA 451 (miR-451) was found to be downregulated in all three comparisons (ND-HD, ND-HDT, and HDT-HD) (Supplementary Table 3). In a previous study, miR-451 levels were higher in participants with non-alcoholic fatty liver disease (NAFLD), which suggests a possible role for this gene in fat metabolism^78,79^. The results of the current study strongly suggest that these genes are critical factors for obesity and could be useful target genes. Additionally, the results of the RT-PCR expression analysis of these nine essential genes (*NUGGC*, *EFR3B*, *RTP4*, *ACAN*, *HOXC4*, *IL17RB*, *SOX13*, *SLC18A2*, and *SOX4*) were similar to the RNA-Seq expression results (Fig. 5), which also validated the high-throughput RNA-Seq analysis.

The gut microbiome can be influenced by diet and can also predict traits such as obesity^80,81^. Considering the importance of the microbiome in diet and obesity, the changes in the microbiome in the HD and HDT groups were studied through 16 s rRNA-based bacterial community analysis. The alpha diversity box plots showed that the gut microbiome diversity was decreased in dogs fed a high-energy diet. A further decrease in diversity was also observed with black ginseng and silkworm treatment (Supplementary Fig. 1). Although previous studies have also shown a decrease in diversity with a high-fat diet^82,83^, more experiments with additional samples (cases) are required to confirm this association.

In the LDA-based analysis, expansion of the class Clostridia was observed in the HD group when compared to the ND group. A similar result was reported in a recent study of a mouse model in which the relative abundance of Clostridia was higher in the HD group than the ND group^84^. Furthermore, a reduction in the family *Lactobacillaceae* was observed in the HD group when compared to the ND group. Previous experiments have also reported a lower abundance of *Lactobacillaceae* in rats fed a high-fed diet^85^. No significant differences in the occurrence of community taxa were observed between the HD group and the HDT group or between the ND group and the HDT group, which may be attributed to these results countering effect of the black ginseng and silkworm supplements against high-energy diet intake.

Although the study was carried out with three subjects/dogs in each group, as the possible triplicate statistical solid significance requires a large number of animals/subjects, therefore, we suggest that the limitation of small sample size, which can be pursued in the extended study in the near future with the more numbers of animals to produce highly reliable findings.

RNA-Seq analysis from adipose tissue can provide precise information about gene expression related to fat metabolism. It can be the sample of choice for the transcriptomic study associated with obesity. Nevertheless, in several studies in humans and mice, the gene expression of blood cells has been used in obesity/high-fat diet-induced obesity, and essential findings were reported^86–90^. In the near future, we also proposed RNA-Seq or single-cell RNA-Seq analysis can be carried out using adipose tissue/cells to study transcriptomic changes in these cells/tissues according to diet.

In this study, we performed comparative transcriptomics and gut microbiome analysis of three groups: beagle dogs with optimal energy intake (ND), high energy intake (HD), and high energy intake and BG + SW supplements (HDT). After 12 weeks of BG + SW supplementation, downregulation of critical factors for obesity and changes in the expression of some essential obesity-related genes were observed. A non-significant decrease in microbiome diversity was observed in the comparison of the HD and HDT groups and significant differences in the occurrence of some taxa in association with a high-fat diet were identified in the HD and ND groups.

## Supplementary Information

Supplementary Information 1.

Supplementary Information 2.
