# Supplementary material for: Effect of black ginseng and silkworm supplementation on obesity, the transcriptome, and the gut microbiome of diet-induced overweight dogs
Source: Sci Rep. 2021 Aug 11;11:16334. doi: 10.1038/s41598-021-95789-8 (PMC8358025; doi:10.1038/s41598-021-95789-8)
Supplement: Supplementary file 1 — Supplementary Information 1. [file 41598_2021_95789_MOESM1_ESM.docx]

Supplement Table 1. The primer sequences used for quantitative PCR analysis.

| **Gene** | **Forward (5' - 3')** | **Reverse (5' - 3')** | **Size (bp)** | **GeneBank accession number** |
| --- | --- | --- | --- | --- |
| NUGGC | ACTCAGCCCATCTATGACC | TCCCATTTCCATCATTTTCTCC | 135 | XM_022410102.1 |
| EFR3B | AAGCACCAGAGAAGGAGAAAG | TAAAGCAACGGATGGCGAAC | 171 | XM_005630212.3 |
| ACAN | TGTCTACCTCTACCCCAACC | TGTGCCACCTTCCTCTTCTC | 115 | NM_001113455.2 |
| RTP4 | TCCAGCTCTTTGGAAGGAAA | TCCCCAGCCATTATGGATTA | 282 | XR_296477.3 |
| HOXC4 | GGAGGTTAGTGAGGGGGAAG | ACCGCCTCTCTGAACTGAAA | 296 | XM_022411242.1 |
| IL17RB | AGCTCCCAAGACCTATTCCC | ACAGAAAGCAGCAGCATCC | 177 | XM_022406600.1 |
| SOX13 | CCATTCCTTTACTCCCTCCC | AAGGCAGGCACCATCAATC | 181 | XM_014111179.2 |
| SLC18A2 | TCCATCCATACACACCAGCC | CCTCGTCTACTTCGTCTTGTC | 174 | XM_022412055.1 |
| SOX4 | TCTCCAACCTGGTCTTCACC | CCGTTCCCTTTTTCTCTTCC | 286 | XM_005640161.3 |

Supplement Table 2. Preprocessing and alignment results of all samples.

| **Sr. No.** | **Sample ID** | **# paired reads** | **Percentage of good reads** | **overall alignment rate** |
| --- | --- | --- | --- | --- |
| 1 | ND1 | 42954582 | 98.58 | 96.52% |
| 2 | ND2 | 36449485 | 98.23 | 95.81% |
| 3 | ND3 | 28887622 | 98.71 | 96.41% |
| 4 | HD1 | 26939465 | 98.13 | 95.48% |
| 5 | HD2 | 32650331 | 98.15 | 94.95% |
| 6 | HD3 | 26079319 | 97.95 | 95.27% |
| 7 | HDT1 | 46703611 | 98.25 | 95.68% |
| 8 | HDT2 | 30433424 | 98.30 | 94.98% |
| 9 | HDT3 | 26913605 | 98.21 | 95.23% |


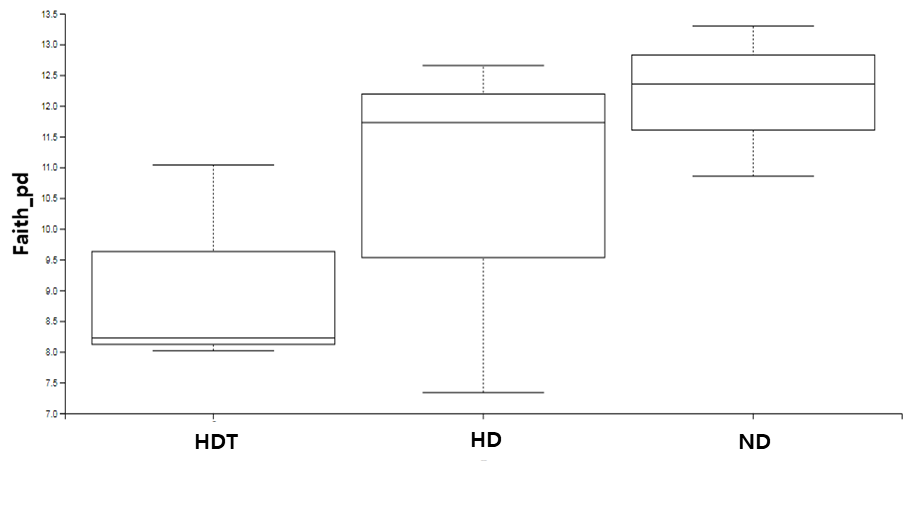


Supplementary Figure 1a. Box plot of Faith phylogeny alpha diversity in different groups.


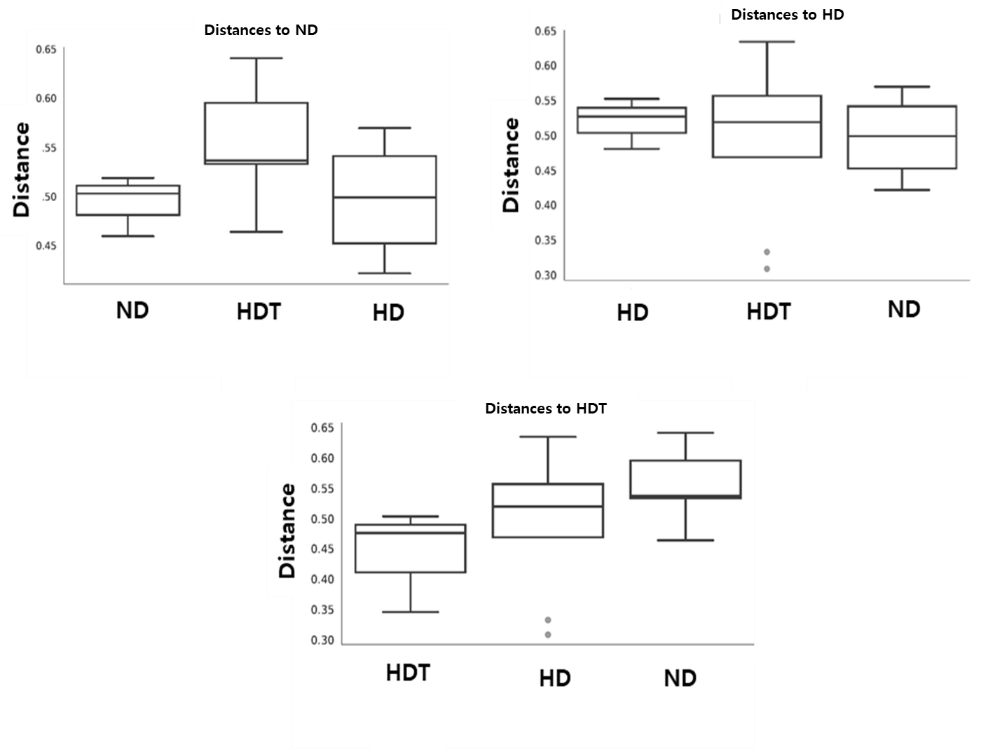

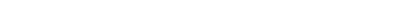


Supplementary Figure 1b. Boxplot showing beta diversity distance reference to each group.


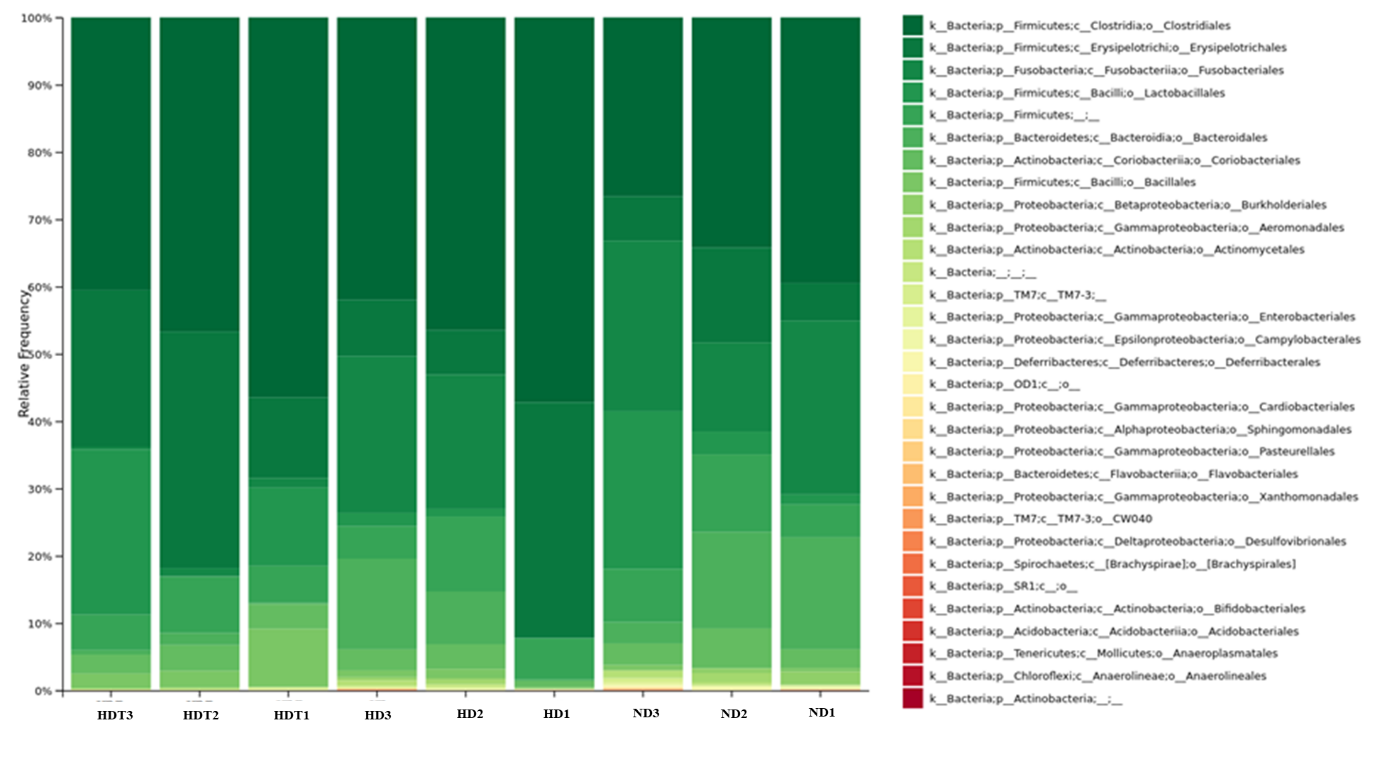


Supplementary Figure 2. Taxonomic annotation of all samples at 4^th^ level in bar plot.
